# Anatomic implantation technique for biventricular support with dual ventricular assist devices

**DOI:** 10.1016/j.xjtc.2026.102382

**Published:** 2026-04-03

**Authors:** Fabian Jimenez Contreras, Ahmet Bilgili, Lindsey M. Brinkley, Johan Van Nisspen, Eric I. Jeng

**Affiliations:** Division of Cardiovascular Surgery, Department of Surgery, University of Florida Health, Gainesville, Fla

**Keywords:** biventricular assist device, mechanical circulatory support

## Abstract

**Objective:**

Bridge-to-heart transplantation with a single HeartMate 3 (HM3) left ventricular assist device has been a proven solution for end-stage heart failure. Some patients require durable right ventricle support for poor baseline biventricular function. We have successfully used dual HM3s in a biventricular configuration to bridge patients to transplant.

**Methods:**

This report discusses, in 8 straightforward yet detailed steps, our technique to implant 2 HM3 devices for dual-chamber support.

**Results:**

In total, 45 successful implants with this configuration of dual HM3 devices have been performed, the greatest reported volume in North America. We supported 1 patient for more than 3 years with durable dual biventricular assist devices, and he was eventually bridged to heart transplantation. More than 50% of our dual biventricular assist device cohort has subsequently received heart transplantation.

**Conclusions:**

Dual HM3 implantation is alternative form of durable biventricular support. Benefits include the length of bridge therapy, the potential for home discharge, enhanced rehabilitation capabilities, and weight loss. This single-center experience demonstrates that this technique can be used to successfully bridge a patient to heart transplantation.

**Figure:**
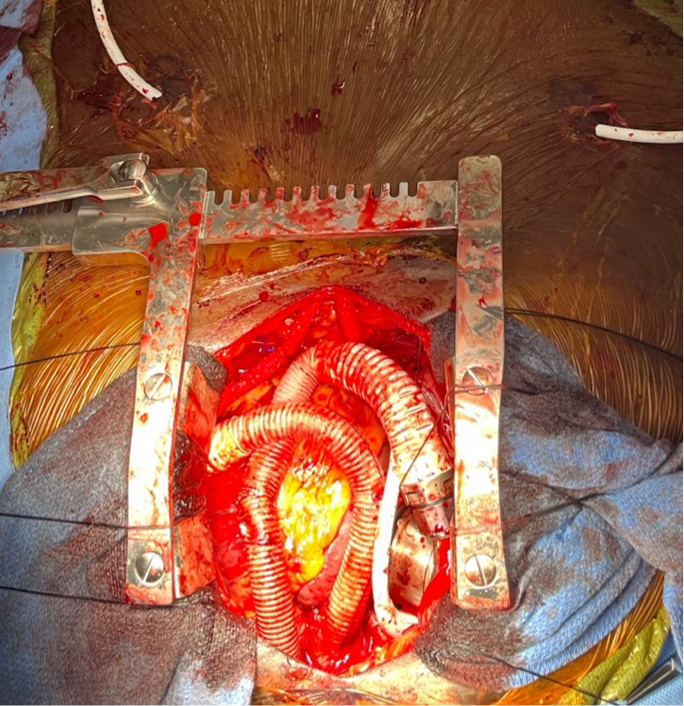
Implanted BiVAD HM3 preceding sternal closure.

**Central Message**

Biventricular support with dual HeartMate 3 devices, as described here, can serve as a bridge to heart transplantation.

**Perspective**

This article will inform mechanical circulatory surgeons worldwide on the technical pearls of BIVAD implantation with a streamlined technique.

Heart transplantation is the gold standard advanced surgical therapy for end-stage heart failure. In recent decades, a proven alternative approach has been the use of a HeartMate 3 (HM3) left ventricular assist device (LVAD) as lifelong therapy and/or to bridge patients to heart transplantation.^1^ This strategy has shown promising results; however, some patients have biventricular failure and require durable right ventricle support.^2^^,^^3^ We have successfully used dual HM3s in a biventricular configuration to bridge these patients to transplantation.^4^ This technique has been used at other centers, often called a “Heart Mate 6.” This report delves into our unique anatomic technique for implanting 2 HM3 devices for dual-chamber ventricular support.

## General Presentation of a Candidate for a Biventricular Assist Device (BiVAD)

A patient between the ages of 18 and 60 years with a history of hypertension, hyperlipidemia, diabetes mellitus, nonischemic cardiomyopathy, heart failure with reduced ejection fraction (ejection fraction 10%), paroxysmal atrial fibrillation, and DM2 is admitted for decompensated heart failure with cardiorenal syndrome requiring a Bumex drip; pushes of Diuril (chlorothiazide), Diamox (acetazolamide), and metolazone; and intra-aortic balloon pump placement for optimization before BiVAD. After a multidisciplinary discussion, the patient is deemed a suitable candidate for durable BiVAD implantation with 2 HM3 devices as destination therapy or bridge to transplantation. The patient does well and is discharged home on postoperative day 21 on acetylsalicylic acid and warfarin with an international normalized ratio goal of 2.5 to 3.5. Common reasons why a patient may not be a candidate for immediate heart transplant at initial evaluation include increased body mass index, a high antibody burden, or expected long period of waiting to find a suitable organ. Institutional review board approval was not required, and patient information is general and not identifiable; thus, consent was waived.

## Detailed Operative Report in 8 Steps

1. A median sternotomy is performed. Before heparin administration, the drivelines for each ventricular assist device are tunneled to the respective side, ensuring maximum length while keeping the Dacron velour interface at the edge of the skin through a purse-string Ethicon VICRYL suture (Figure 1). We have not encountered any anatomic abnormalities that have caused us to deviate from the following protocol.

**Figure 1:**
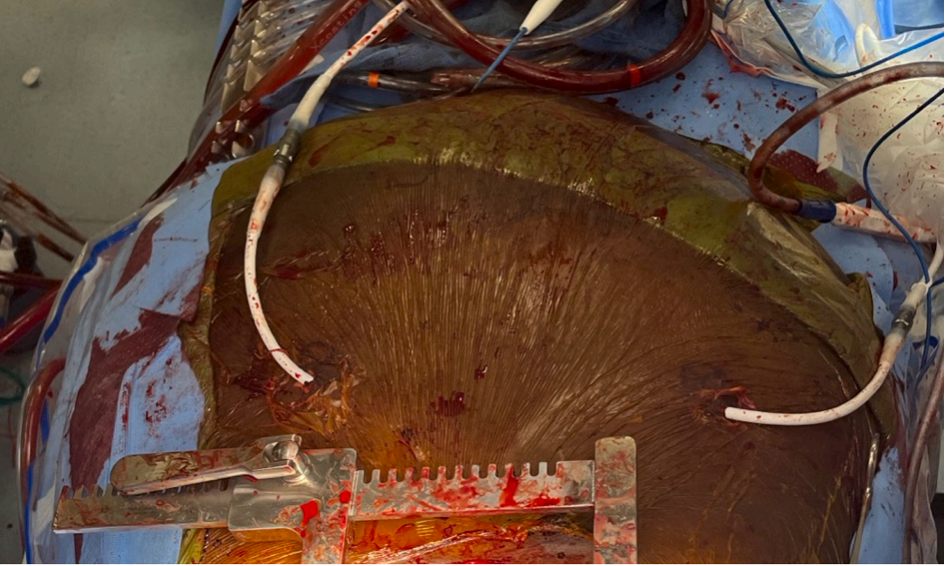
Bilateral tunnel drivelines are shown.
2. The canulation strategy includes central aortic canulation, bicaval central venous cannula, and a left ventricular vent via the right superior pulmonary vein. Of note, a percutaneous femoral cannula can be used as ICV to declutter the surgical field if preferred.
3. A pulmonary arteriotomy is created and rounded using a 4.0-mm punch for the right ventricular assist device (RVAD) outflow site. An 8-mm Gelweave graft (Terumo Aortic) is sewn to the distal main pulmonary artery at the level of the pulmonary artery bifurcation. A partial occlusion clamp or drop sucker can be placed in the pulmonary artery to help facilitate visualization. The anastomosis is completed with a 3-0 RB-1 (Ethicon) or similar suture.
4. The heart is then elevated, with 4 to 5 laparotomy sponges, to expose the left ventricular apex for the LVAD inflow site. The cannulation site is selected just parallel to the left anterior descending artery just inferior and lateral to the true apex. Twelve circumferential 2-0 Ti-Cron sutures (Medtronic) with large homemade pledgets are placed equidistantly around the sewing ring. We use 4 colored sutures in the 4 cardinal directions, and then 2 white sutures between each of colored sutures, to total 12; this is our preference to ensure hemostasis. The left ventricular apex is cored manually with a no. 11/15 blade, and endocardial myomectomy is performed to optimize inflow (Figure 2). We prefer this method compared with using the included HM3 coring tool because there is less risk of injuring the ventricular septum.

**Figure 2:**
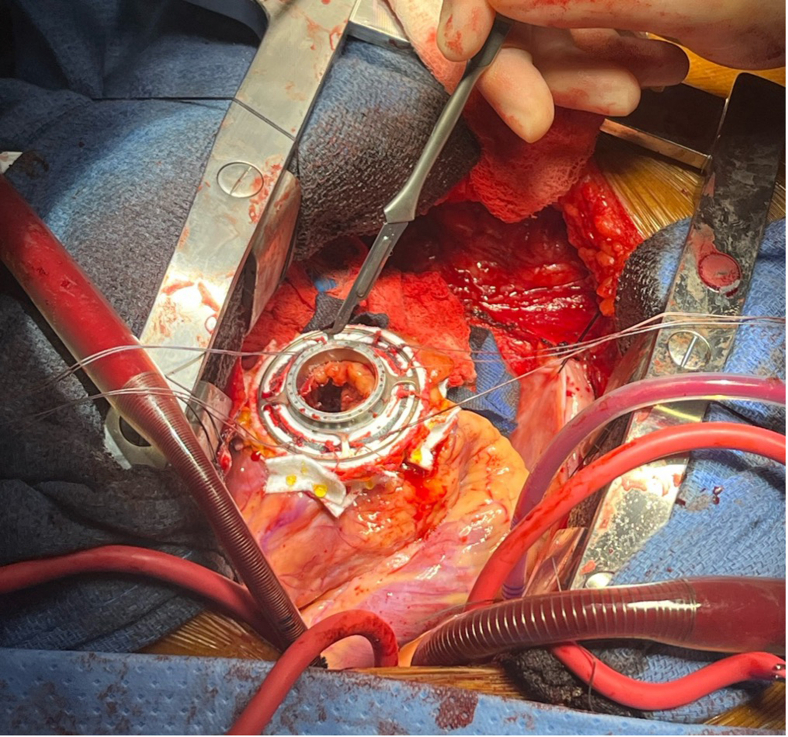
The left ventricular apex is cored manually with a no. 15 blade.
5. We generally select a site at least 1 cm above the atrioventricular groove and as far away as possible lateral from the atrial septum while still being on the anterior face of the right atrium for the RVAD inflow site. A ring of 10 felt donuts, held together with the provided Coring knife with protected plastic covering. A 3-0 PROLENE suture (Ethicon) is run around the felt ring aggregate circumferentially so as to keep the cored felt inner diameter consistent in size (Figures 3 and 4). The 10 felt donuts provide just enough spacing to prevent atrial suction. Twelve small pledgetted sutures are placed circumferentially from inside out through the atrium and the sewing cuff/donut aggregate (Figure 5). During this portion, placing the Coring knife with protected plastic covering prevents ovalization of the aggregate sewing cuff and enables easier securement of the RVAD once the sutures are tied.

**Figure 3:**
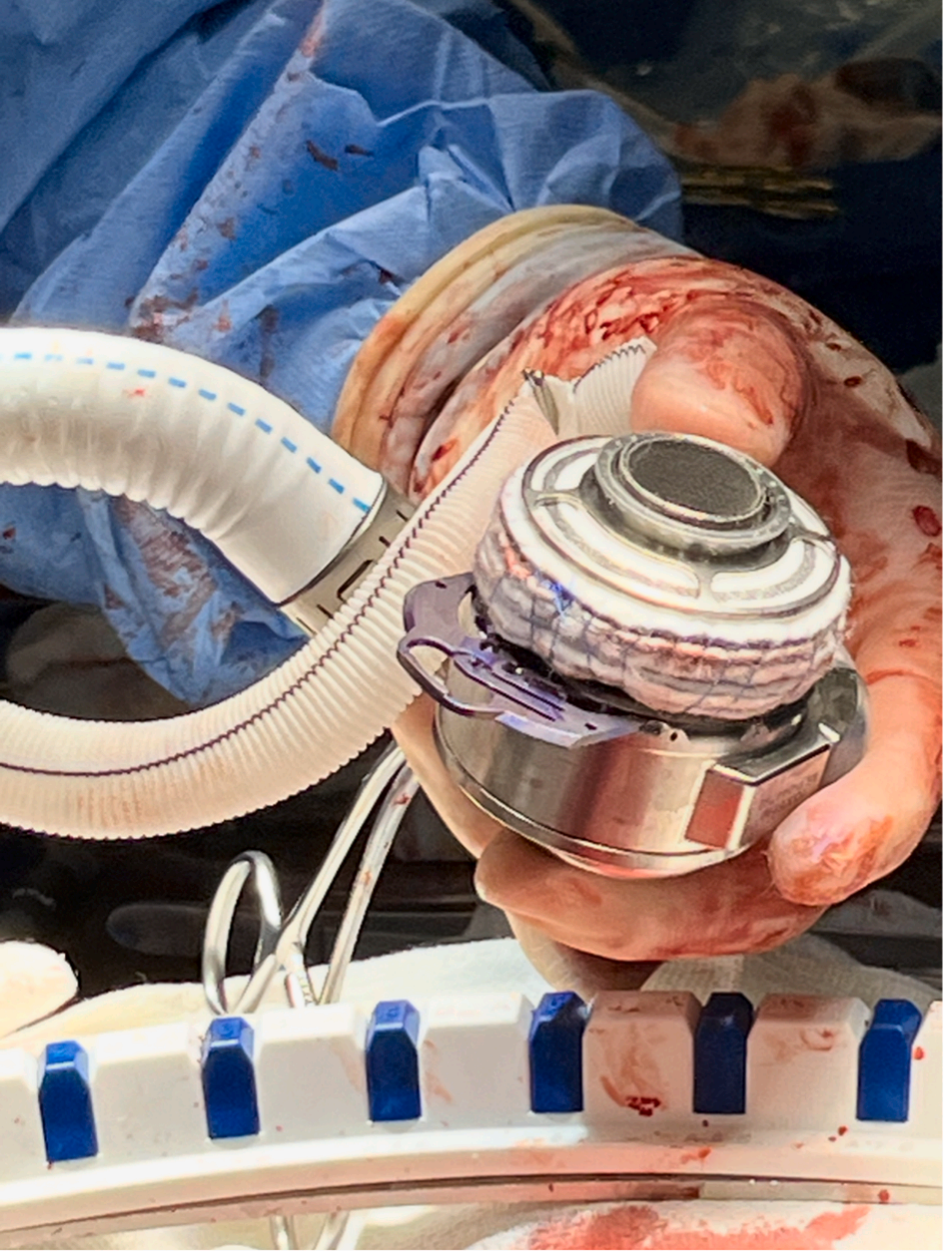
Ten felt donuts are used to make a cuff for the right ventricular assist device, placed in a nonlocking position to aid in display.

**Figure 4:**
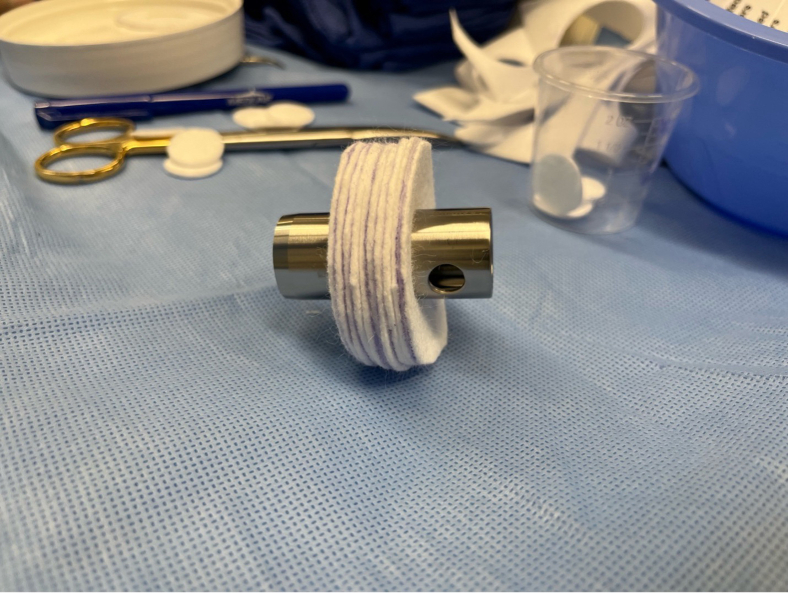
A ventricle cutter is used to hold right ventricular assist device felt rings while being secured with a running PROLENE suture.

**Figure 5:**
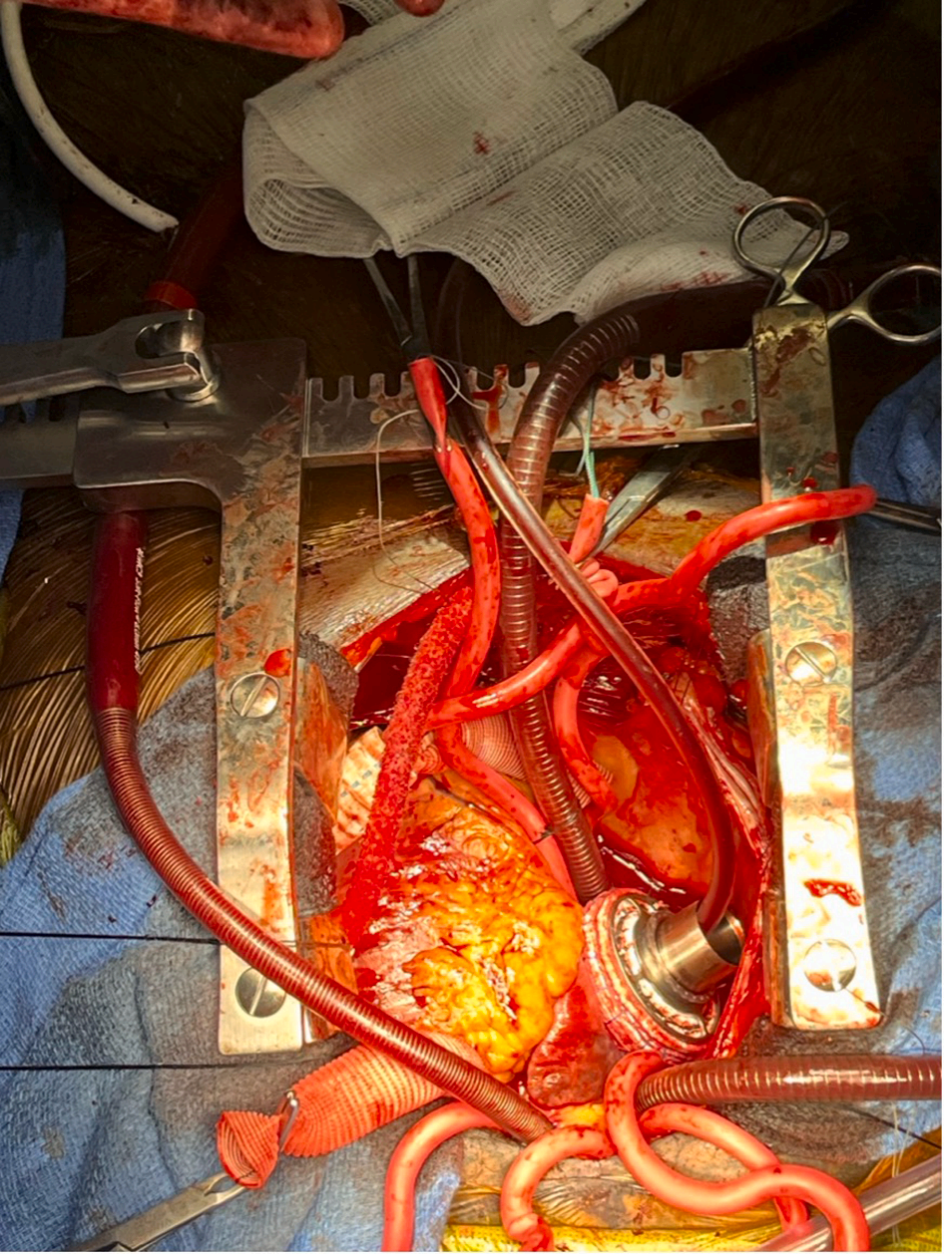
Position of the right ventricular assist device inflow site on the right atrium is shown. The selection should be performed with care taken to avoid the atrial septum.
6. A 14-mm to 8-mm graft-to-graft anastomosis is performed to complete the RVAD outflow. The graft is also deaired and the RVAD is initiated at low speed (4000 rpm) (Figure 6).

**Figure 6:**
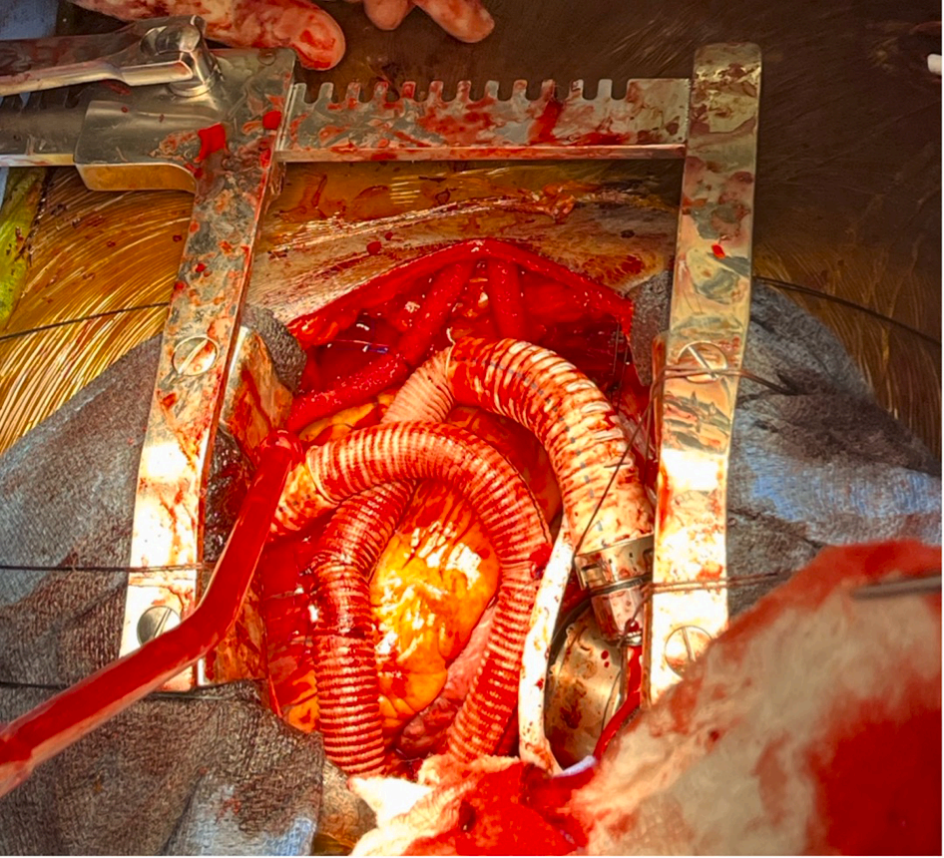
End-to-end anastomosis for right ventricular assist device outflow tract and final position of biventricular assist device configuration are shown.

**Figure 7:**
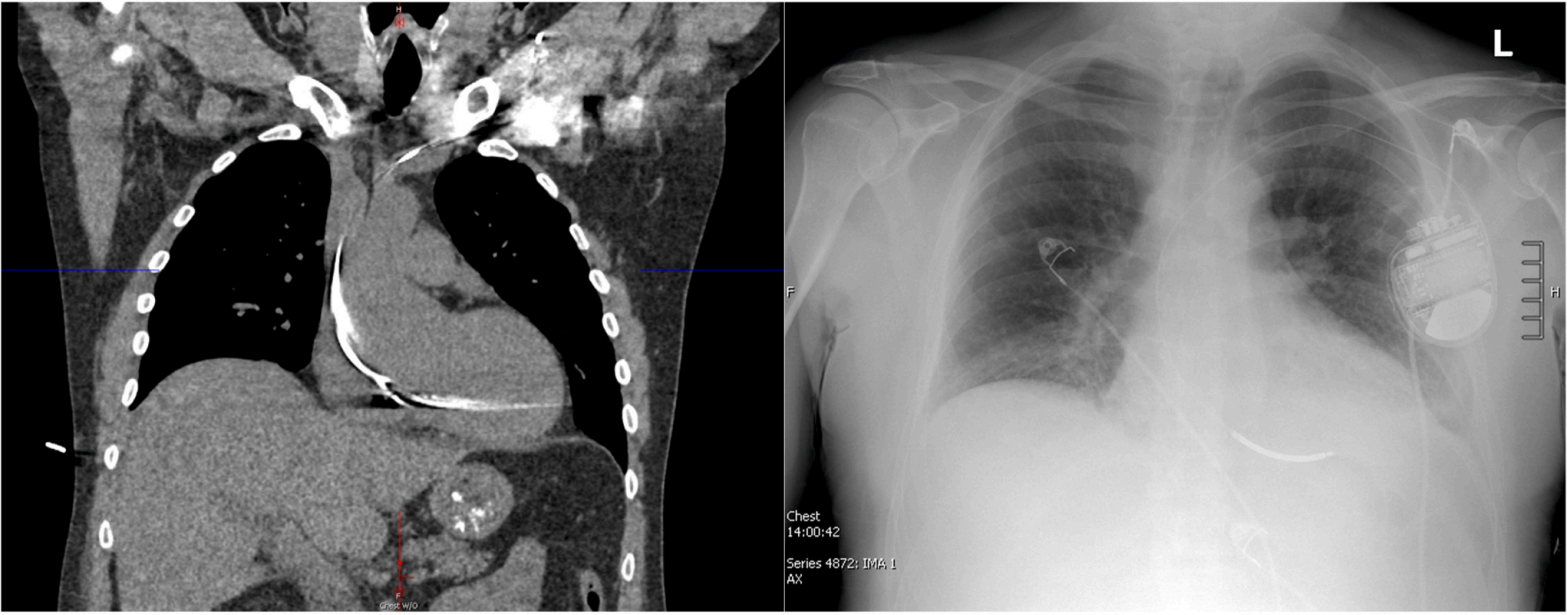
Preimplant computed tomography scan (*left*) and radiograph of the chest (*right*).

**Figure 8:**
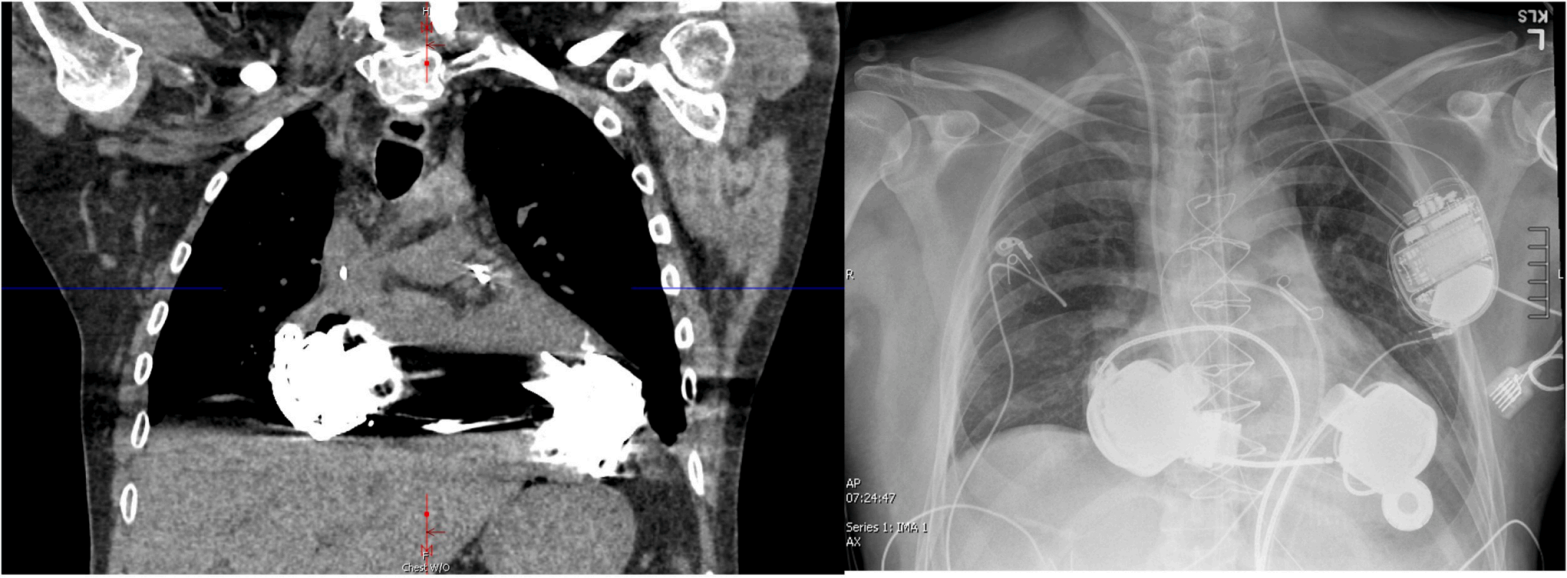
Postimplant computed tomography scan (*left*) and radiograph of the chest (*right*).
7. The LVAD outflow graft is trimmed to an appropriate length. A partial occlusion clamp is placed on the ascending aorta, and a rounded aortotomy is created. A circumferential anastomosis is performed with a running 3-0 PROLENE RB 1. The aorta is deaired and the LVAD also is initiated at 4000 rpm. We wean the patient off cardiopulmonary bypass and bring them up on biventricular flow with the goal of achieving full flow in both VADs. We err on the side of greater flow in the LVAD to allow for slightly more volume in the right heart to Prevent suction events. We balance the speeds on both VADs using the ventricular septum on transesophageal echocardiography to avoid bowing.
8. After sternal closure, 3 separate no. 5 ETHIBOND sutures (Ethicon) are used to suspend the RVAD chassis in an optimal position relative to the chest wall with distinct fixation points at the sternum around the ribs. This prevents suction to the atrial septum, which is a common problem if this step is omitted.

## Comment

Dual HM3 implantation offers a promising alternative to other biventricular support methods, such as venoarterial extracorporeal membrane oxygenation, for select patients with advanced heart failure. Key advantages include the potential for home discharge, enhanced rehabilitation capabilities, and weight loss, which can make patients eligible for heart transplantation.^3^^,^^5^

At our institution, we have successfully implanted 45 patients using this configuration, achieving the greatest volume, to our knowledge, in North America. These implants were preformed between 2012 and 2025. The median duration of postoperative hospital length of stay was 40 days. In total, 31% (14/45) of all patients were able to leave the hospital before transplantation. The median of duration of time between BiVAD implant and transplantation was 30 days. We have experienced low rates of complications: significant bleeding at 22% (10/45), infection at 6% (3/45), and stroke at 2% (1/45). Notably, we supported 1 patient for more than 300 days with a dual HM3 BiVAD, who subsequently underwent transplantation.^4^ Approximately 50% of patients initially deemed nontransplant candidates who receive the dual HM3 BiVAD at our center successfully undergo heart transplantation. This success should motivate other centers to explore this approach as a bridge to transplantation for their patients.

## Conclusions

We introduce an innovative approach using dual HM3s to deliver biventricular support to patients who cannot withstand a single HM3 as a standalone LVAD because of a severely compromised right ventricle. This method has proven effective in bridging patients to heart transplantation.

## Conflict of Interest Statement

The authors reported no conflicts of interest.

The *Journal* policy requires editors and reviewers to disclose conflicts of interest and to decline handling or reviewing manuscripts for which they may have a conflict of interest. The editors and reviewers of this article have no conflicts of interest.
